# Supplementary material for: Deleterious heteroplasmic mitochondrial mutations are associated with an increased risk of overall and cancer-specific mortality
Source: Nat Commun. 2023 Sep 30;14:6113. doi: 10.1038/s41467-023-41785-7 (PMC10542802; doi:10.1038/s41467-023-41785-7)
Supplement: Supplementary file 1 — Supplementary files [file 41467_2023_41785_MOESM1_ESM.pdf]

Table of Contents:

Supplementary Methods – Page 2

Supplementary Table 1. Number of heteroplasmic variants shared by related individuals. – Page 3

Supplementary Table 2. Hazard ratios (95% confidence intervals) for all-cause mortality by variant allele fraction (VAF) and MLC score sum (MSS). – Page 3

Supplementary Table 3. Adjusted hazard ratios for the associations of heteroplasmy count, MSS, and each outcome. – Page 3

Supplementary Table 4. ICD-10 codes for each cause of death. – Page 4

Supplementary Table 5. ICD-10 and ICD-9 codes for each type of cancer. – Page 5

Supplementary Figure 1. Flowchart. – Page 7

Supplementary Figure 2. Data QC and participant exclusion criteria. – Page 8

Supplementary Figure 3. Distribution of mitochondrial DNA copy number by heteroplasmy count. – Page 9

Supplementary Figure 4. Hazard ratios (95% confidence intervals) for all-cause mortality by heteroplasmy count adjusted for mitochondrial mutation annotations. – Page 10

Supplementary Figure 5. Hazard ratios (95% confidence intervals) for all-cause mortality by heteroplasmy count, presence of nonsynonymous mutation, and MLC score sum (MSS). Page – 11

Supplementary Figure 6. Meta-analysis of the association between mitochondrial heteroplasmy and all-cause mortality. Page – 12

Supplementary Figure 7. Hazard ratios (95% confidence intervals) for mitochondrial heteroplasmy and all-cause mortality by self-identified race/ethnic background in the UK Biobank. – Page 13

Supplementary Figure 8. Meta-analysis of the association between mitochondrial heteroplasmy and all-cause mortality by race. – Page 14

Supplementary Figure 9. Comparison of hazard ratios at different mitochondrial heteroplasmy and mtDNA-CN thresholds. – Page 15

Supplementary Figure 10. Heatmap displaying the significance (-log10(P value)) of the difference between effect estimates for MSS and ICD 10 codes for cancer ("Chapter II Neoplasms") and hematological diseases ("Chapter III Disease of the blood and blood-forming organs and certain disorders involving the immune mechanism") by mtDNA region/complex. – Page 16

Supplementary Methods:

Statistical comparison of beta estimates of PHESANT analysis across complexes

PHESANT beta estimates and p values were used to calculate standard errors (SE) by first converting p values to z-scores using the qnorm function in R, accounting for the 2-sided nature of the p values. The standard errors were then calculated as the absolute value of the beta estimates divided by the z-scores.

To compare beta estimates across complexes, beta estimates were first transformed to a z-scale using the following equation: beta` = 0.5 ln [(1 + beta) / (1 – beta)]. Significance was then determined by applying the pnorm function to the difference between the z-scaled estimates divided by the pooled standard errors, which follows a z-score distribution: $beta_1$` – $beta_2$` / sqrt(SE_1^2 + SE_2^2)

Supplementary Table 1. Number of heteroplasmic variants shared by related individuals.

	Number of variants identified	Number of variants shared by the pair (%)
**Monozygotic twins (28 pairs)**	12	7 (58.3%)
**Mother/child (760 pairs)**	352	105 (29.8%)
**Full siblings (3,657 pairs)**	1372	403 (29.4%)

Supplementary Table 2. Hazard ratios (95% confidence intervals) for all-cause mortality by variant allele fraction (VAF) and MLC score sum (MSS).

	VAF only	MSS only	Both VAF and MSS	
	VAF (per 10%)	MSS (per 1-unit)	VAF	MSS
**Randomly selected heteroplasmic SNV**	0.99 (0.98, 1.01)	1.32 (1.21, 1.44)	0.99 (0.98, 1.01)	1.30 (1.20, 1.41)
**Heteroplasmic SNV with the largest MLC score**	0.99 (0.98, 1.00)	1.32 (1.21, 1.44)	1.00 (0.99, 1.01)	1.31 (1.20, 1.42)
**Singleton**	0.99 (0.98, 1.00)	1.40 (1.22, 1.61)	0.99 (0.98, 1.01)	1.33 (1.17, 1.51)

*All models are adjusted for age, sex, smoking status, and stratified by center.

Supplementary Table 3. Adjusted hazard ratios for the associations of heteroplasmy count, MSS, and each outcome.

	Model 1	Model 2
**Heteroplasmy count**		
**Overall mortality**	1.05 (1.03, 1.08)	1.05 (1.02, 1.07)
**Any cancer**	1.03 (1.01, 1.05)	1.02 (1.00, 1.04)
**Hematologic cancer**	1.23 (1.15, 1.32)	1.21 (1.13, 1.29)
**Lymphoma**	1.10 (1.00, 1.22)	1.10 (1.99, 1.22)
**Leukemia**	1.54 (1.38, 1.71)	1.48 (1.33, 1.65)
**MSS**		
**Overall mortality**	1.11 (1.05, 1.18)	1.11 (1.04, 1.17)
**Any cancer**	1.11 (1.05, 1.18)	1.10 (1.04, 1.17)
**Hematologic cancer**	1.89 (1.59, 2.25)	1.81 (1.51, 2.15)
**Lymphoma**	1.45 (1.09, 1.92)	1.41 (1.06, 1.88)
**Leukemia**	3.42 (2.71, 4.33)	3.11 (2.45, 3.96)

Model 1: adjusted for age (restricted cubic splines with 4 degrees of freedom), sex, smoking status, alcohol intake, WBC count, and haplogroup; Model 2: Model 1 + clonal hematopoiesis of indeterminate potential.

Supplementary Table 4. ICD-10 codes for each cause of death.

Cause of death	ICD-10 (Version 2010)
Infections	A00 – B99, L00 – L08
Neoplasms	C00 – D48
Cancers	C00 – C97
Solid cancers	C00 – C80, C97
Hematologic cancers	C81 – C96
Benign neoplasms	D00 – D48
Benign diseases of the blood	D50 – D89
Endocrine disorders	E00 – E90
Mental and behavioral disorders	F00 – F89
Neurological disorders	G00 – G99
Circulatory disorders	I05 – I89
Respiratory disorders	J09 – J99
Digestive disorders	K20 – K93
Genitourinary disorders	N00 – N98
COVID-19	U07
External causes, including accidents, injuries, poisoning, and drugs and biological substances	V01 – Y89
Accidents	V01 – V49
Intentional self-harm	X60 – X84

Supplementary Table 5. ICD-10 and ICD-9 codes for each type of cancer.

Type of cancer	ICD-10 (Version 2010)	ICD-9
Lip, oral cavity, and pharynx	C00 – C14	140 – 149
Digestive organs	C15 – C26	150 – 157, 159
Respiratory and intrathoracic organs (lung)	C30 – C39	160 – 165
Bone and articular cartilage	C40, C41	170
Skin (Malignant melanoma)	C43	172
Mesothelial and soft tissue	C45 – C49	158, 171, 176
Breast (in women)	C50	174, 175
Female genital organs	C51 – C58	179 – 184
Male genital organs	C60 – C63	185 – 187
Urinary tract	C64 – C68	188, 189
Eye, brain, and other parts of central nervous system	C69 – C72	190 – 192
Thyroid and other endocrine glands	C73 – C75	193, 194
Ill-defined, secondary, and unspecified sites	C76 - C80	195 – 199
Lymphoid, hematopoietic, and related tissue	C81 – C96	200 – 208
Lymphoma	C81 – C86	200 – 202
Leukemia	C91 – C95	204 – 208

Supplementary Figures

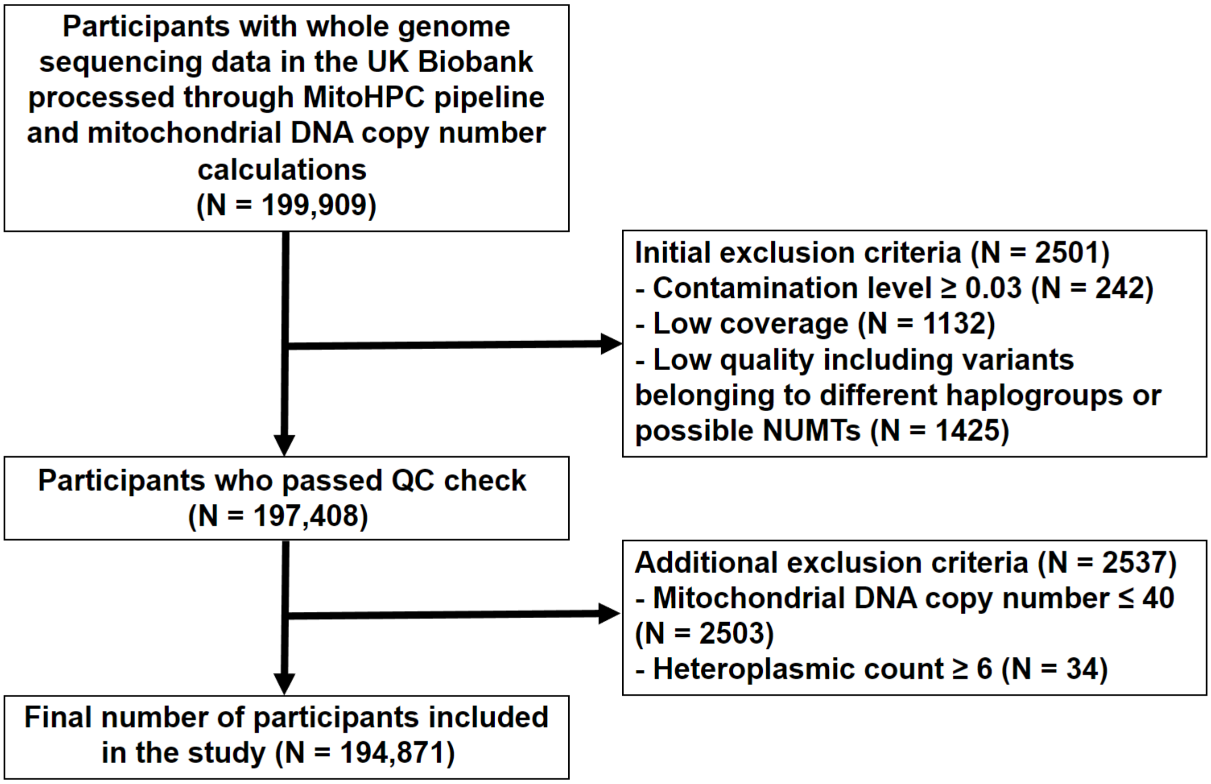

Supplementary Figure 1. Flowchart. Of the 199,919 samples with outputs from MitoHPC variant calling, we calculated mtDNA copy number (mtDNA-CN) on 199,909 samples. We first excluded 2501 participants who did not meet QC criteria (potential mitochondrial contamination, low minimum base coverage, low mean base coverage, 2 or more variants belonging to a different mitochondrial haplogroup, and multiple variants predicted to be Nuclear-encoded mitochondrial sequences [NUMTs]). We further removed participants with mtDNA-CN less than 40 or participants with a heteroplasmic count above 5. 194,871 participants were included in the study for downstream analysis

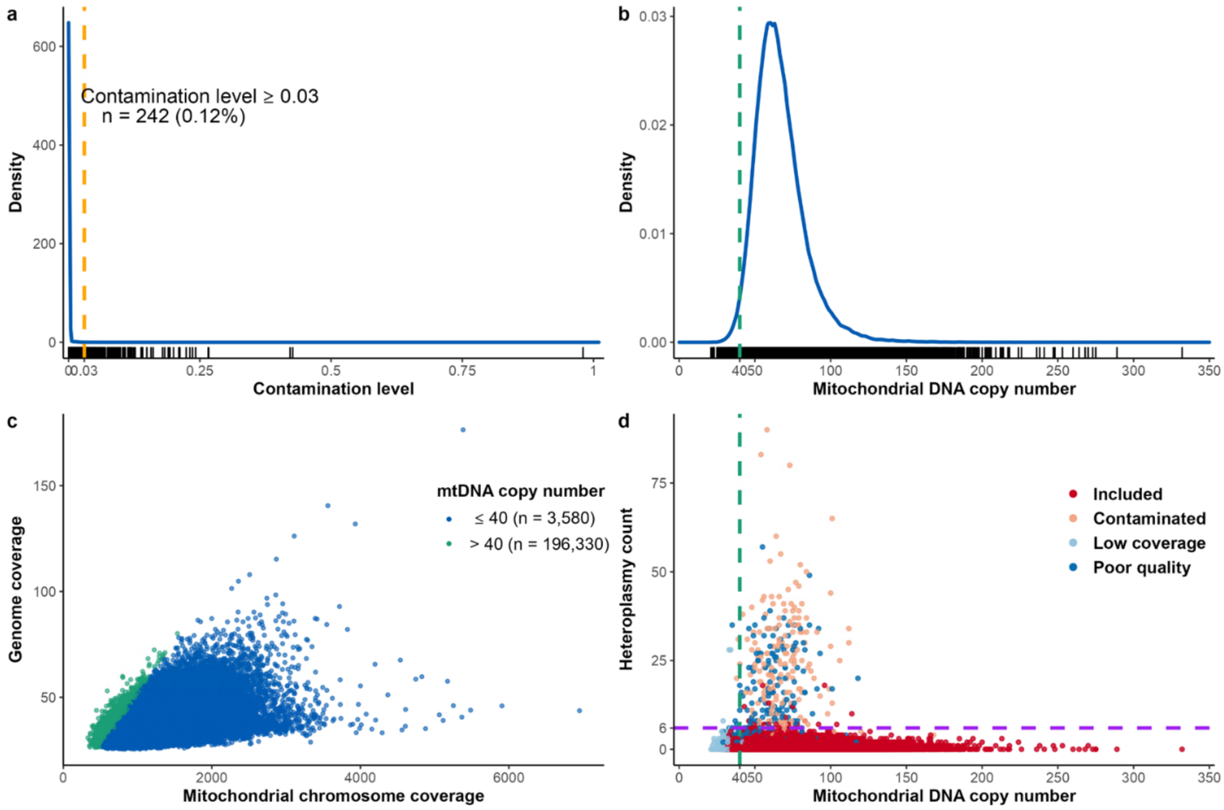

Supplementary Figure 2. Data QC and participant exclusion criteria. (a) Contamination level for each sample (participant) plotted in a density plot. Each sample is represented by a vertical line in the rug plot along the X-axis. Orange dashed line indicates a contamination level of 3%. Samples with 3% or more contamination were excluded from analysis (n = 242). (b) Density plot of mitochondrial DNA copy number (mtDNA-CN) for all samples. Each sample is represented by a vertical line in the rug plot along the x-axis. Green dashed line indicates the cutoff (mtDNA-CN ≤ 40) for low mtDNA-CN samples (n = 3,580). (c) A scatter plot of mitochondrial chromosome coverage versus genome coverage stratified by mtDNA-CN cutoff. Samples with green dots have low mtDNA-CN and were excluded from analysis. (d) A scatter plot of mtDNA-CN and heteroplasmy count per sample in the entire dataset. Red dots are samples there were included in the study. Orange dots indicate samples that did not pass contamination threshold. Light blue dots indicate samples that were excluded due to low mitochondrial coverage defined as mean coverage less than 500 or minimum coverage less than 100. Dark blue dots indicate samples that were excluded that had other indicators of inadequate quality such as single nucleotide variants (SNVs) from a different haplogroup than the MitoHPC-identified haplogroup, or multiple SNVs matching known NUMTs (nuclear encoded mitochondrial sequences). The vertical green dashed line indicates the threshold for CN (≤ 40) at which samples were excluded from downstream analysis. The horizontal purple line indicates participants with heteroplasmy count of 6 and above. These high heteroplasmy count participants were also excluded from analysis.

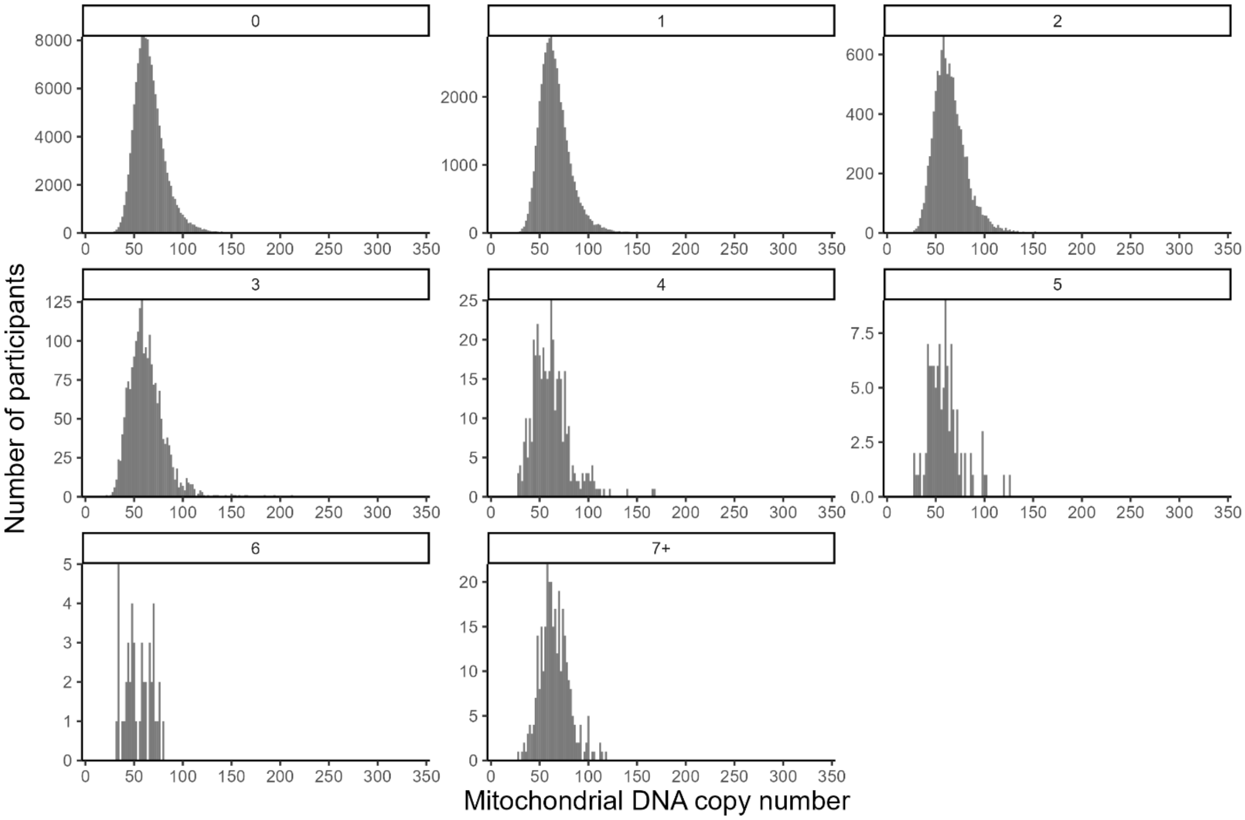

Supplementary Figure 3. Distribution of mitochondrial DNA copy number by heteroplasmy count. Histograms of mtDNA copy number by heteroplasmy count.

a

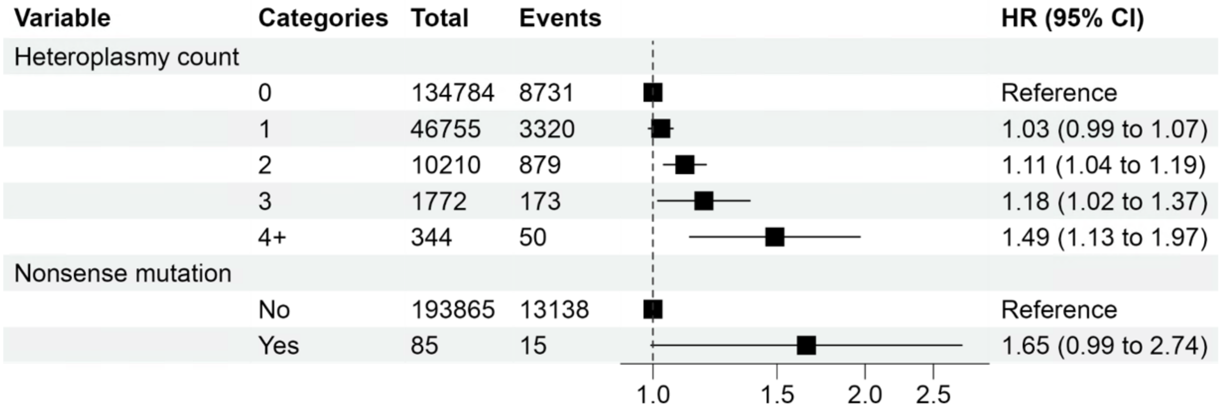

b

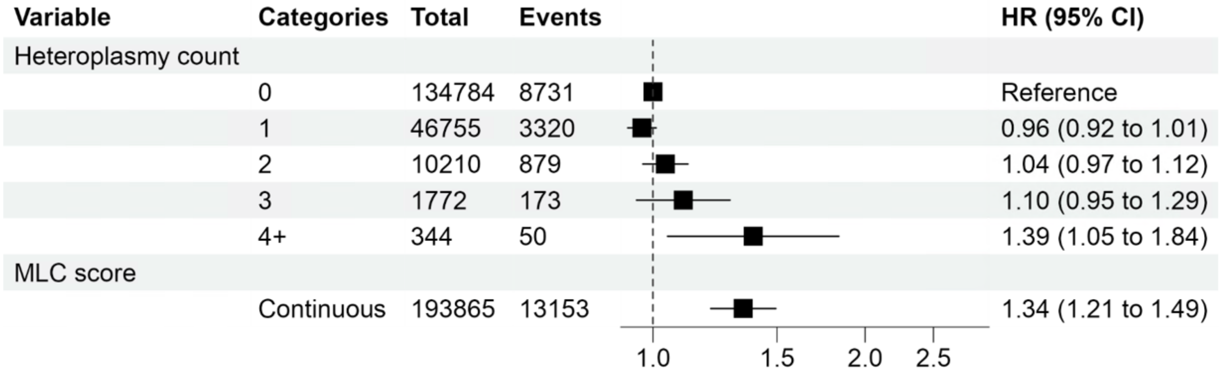

Supplementary Figure 4. Hazard ratios (95% confidence intervals) for all-cause mortality by heteroplasmy count adjusted for nonsense mutation and MLC score. Hazard ratios for all-cause mortality by heteroplasmy count were adjusted for **(a)** nonsense mutation and for **(b)** MLC score. The associations were estimated from Cox proportional hazards models stratified by assessment center and adjusted for age, sex, and smoking status (never, former, or current smoker). The MLC score was generated from a randomly selected heteroplasmic SNV from each participant.

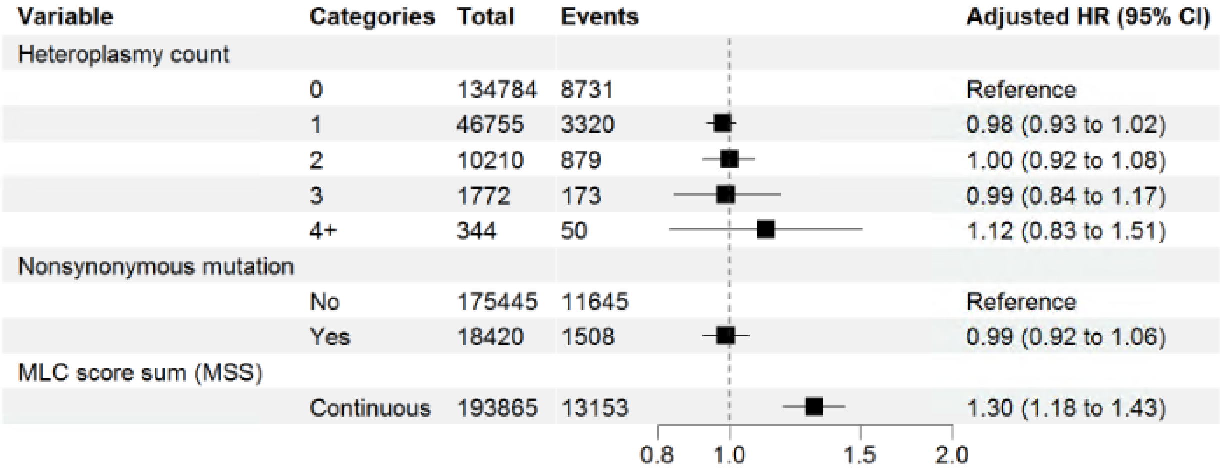

Supplementary Figure 5. Hazard ratios (95% confidence intervals) for all-cause mortality by heteroplasmy count, presence of nonsynonymous mutation, and MLC score sum (MSS). Hazard ratios for all-cause mortality were estimated for heteroplasmy count, presence of nonsynonymous mutation, and MSS from a Cox proportional hazards model stratified by assessment center and adjusted for age, sex, and smoking status (never, former, or current smoker).

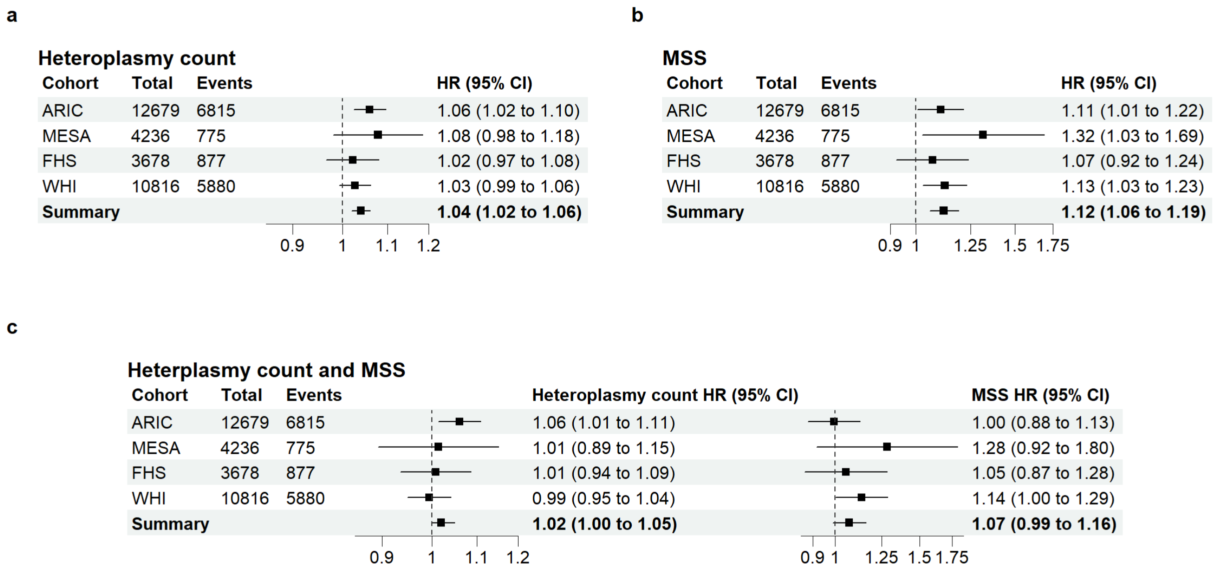

Supplementary Figure 6. Meta-analysis of the association between mitochondrial heteroplasmy and all-cause mortality. Hazard ratios (95% confidence intervals) for all-cause mortality by heteroplasmy count (a), MLC score sum (MSS) (b), and both heteroplasmy count and MSS (c) were estimated in each cohort, using Cox proportional hazards models adjusting for age, sex, and smoking status. For Women's Health Initiative (WHI) study, sex was not included in the model because all participants were women. Pooled hazard ratios and corresponding 95% confidence intervals were estimated using fixed-effects meta-analysis. Abbreviations: ARIC, Atherosclerosis Risk in Communities; FHS, Framingham Heart Study; MESA, Multi-Ethnic Study of Atherosclerosis. The numbers of participants and events reflect the number included in the regression analysis.

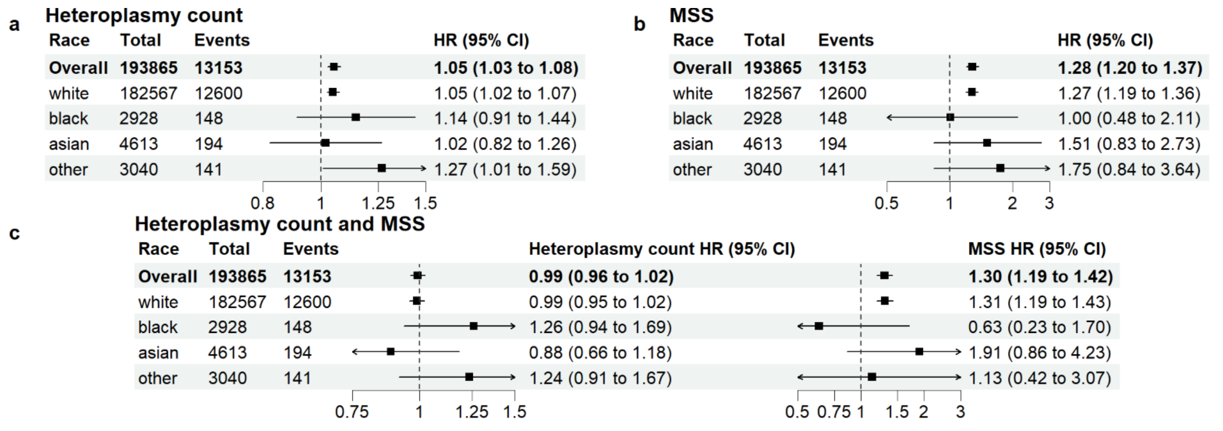

Supplementary Figure 7. Hazard ratios (95% confidence intervals) for mitochondrial heteroplasmy and all-cause mortality by self-identified race/ethnic background in the UK Biobank. Hazard ratios for all-cause mortality by heteroplasmy count (**a**) and MLC score sum (MSS) (**b**) were estimated from Cox proportional hazards models stratified by assessment center and adjusted for age, sex, and smoking status (never, former, or current smoker). In addition, heteroplasmy count and MSS were further adjusted for each other (**c**).

a

Heteroplasmy count

Group	Cohort	Total	Events	HR (95% CI)
White				
	ARIC	9953	5281	1.06 (1.02 to 1.10)
	MESA	1744	331	1.02 (0.89 to 1.17)
	FHS	3678	877	1.02 (0.97 to 1.08)
	WHI	8823	5053	1.03 (0.99 to 1.07)
	UKB	182567	12600	1.05 (1.02 to 1.07)
	**Summary**			**1.04 (1.03 to 1.06)**
Black				
	ARIC	2726	1534	1.07 (0.99 to 1.15)
	MESA	984	202	1.04 (0.84 to 1.28)
	WHI	1405	597	0.96 (0.86 to 1.08)
	UKB	2928	148	1.15 (0.91 to 1.44)
	**Summary**			**1.04 (0.98 to 1.10)**
Hispanic				
	MESA	947	155	1.11 (0.90 to 1.37)
	WHI	304	105	0.91 (0.68 to 1.22)
	**Summary**			**1.03 (0.87 to 1.23)**
Asian				
	MESA	561	87	1.35 (1.07 to 1.72)
	WHI	201	85	1.36 (1.03 to 1.79)
	UKB	4613	194	1.02 (0.82 to 1.26)
	**Summary**			**1.21 (1.05 to 1.39)**

b

MSS

Group	Cohort	Total	Events	HR (95% CI)
White				
	ARIC	9953	5281	1.13 (1.02 to 1.26)
	MESA	1744	331	1.30 (0.91 to 1.87)
	FHS	3678	877	1.07 (0.92 to 1.24)
	WHI	8823	5053	1.14 (1.03 to 1.25)
	UKB	182567	12600	1.27 (1.19 to 1.36)
	**Summary**			**1.19 (1.14 to 1.25)**
Black				
	ARIC	2726	1534	1.00 (0.82 to 1.22)
	MESA	984	202	0.89 (0.50 to 1.59)
	WHI	1405	597	0.96 (0.69 to 1.34)
	UKB	2928	148	1.01 (0.48 to 2.11)
	**Summary**			**0.98 (0.84 to 1.15)**
Hispanic				
	MESA	947	155	1.58 (0.91 to 2.73)
	WHI	304	105	0.35 (0.11 to 1.16)
	**Summary**			**1.21 (0.74 to 2.00)**
Asian				
	MESA	561	87	2.52 (1.28 to 4.97)
	WHI	201	85	1.62 (0.80 to 3.26)
	UKB	4613	194	1.51 (0.83 to 2.73)
	**Summary**			**1.80 (1.24 to 2.63)**

c

Heterplasmy count and MSS

Group	Cohort	Total	Events	Heteroplasmy count HR (95% CI)	MSS HR (95% CI)
White					
	ARIC	9953	5281	1.05 (1.00 to 1.10)	1.04 (0.90 to 1.20)
	MESA	1744	331	0.92 (0.76 to 1.11)	1.52 (0.92 to 2.51)
	FHS	3678	877	1.01 (0.94 to 1.09)	1.05 (0.87 to 1.28)
	WHI	8823	5053	0.99 (0.94 to 1.04)	1.15 (1.01 to 1.31)
	UKB	182567	12600	0.99 (0.95 to 1.02)	1.31 (1.19 to 1.43)
	**Summary**			**1.00 (0.98 to 1.02)**	**1.19 (1.12 to 1.27)**
Black					
	ARIC	2726	1534	1.12 (1.02 to 1.23)	0.83 (0.64 to 1.07)
	MESA	984	202	1.11 (0.85 to 1.45)	0.74 (0.35 to 1.55)
	WHI	1405	597	0.95 (0.81 to 1.11)	1.07 (0.68 to 1.68)
	UKB	2928	148	1.26 (0.94 to 1.69)	0.63 (0.23 to 1.70)
	**Summary**			**1.08 (1.00 to 1.17)**	**0.86 (0.69 to 1.06)**
Hispanic					
	MESA	947	155	0.98 (0.73 to 1.32)	1.62 (0.76 to 3.47)
	WHI	304	105	1.09 (0.77 to 1.54)	0.29 (0.07 to 1.21)
	**Summary**			**1.03 (0.82 to 1.29)**	**1.11 (0.57 to 2.16)**
Asian					
	MESA	561	87	1.18 (0.84 to 1.67)	1.79 (0.65 to 4.89)
	WHI	201	85	1.44 (0.94 to 2.20)	0.82 (0.27 to 2.52)
	UKB	4613	194	0.88 (0.66 to 1.18)	1.91 (0.86 to 4.23)
	**Summary**			**1.08 (0.89 to 1.31)**	**1.53 (0.89 to 2.64)**

Supplementary Figure 8. Meta-analysis of the association between mitochondrial heteroplasmy and all-cause mortality by race. Hazard ratios (95% confidence intervals) for all-cause mortality by heteroplasmy count (a), MLC score sum (MSS) (b), and both heteroplasmy count and MSS (c) were estimated in each cohort, using Cox proportional hazards models adjusting for age, sex, and smoking status. For Women's Health Initiative (WHI) study, sex was not included in the model because all participants were women. Pooled hazard ratios and corresponding 95% confidence intervals were estimated using fixed-effects meta-analysis. Abbreviations: ARIC, Atherosclerosis Risk in Communities; FHS, Framingham Heart Study; MESA, Multi-Ethnic Study of Atherosclerosis; UKB, UK Biobank. The numbers of participants and events reflect the number included in the regression analysis.

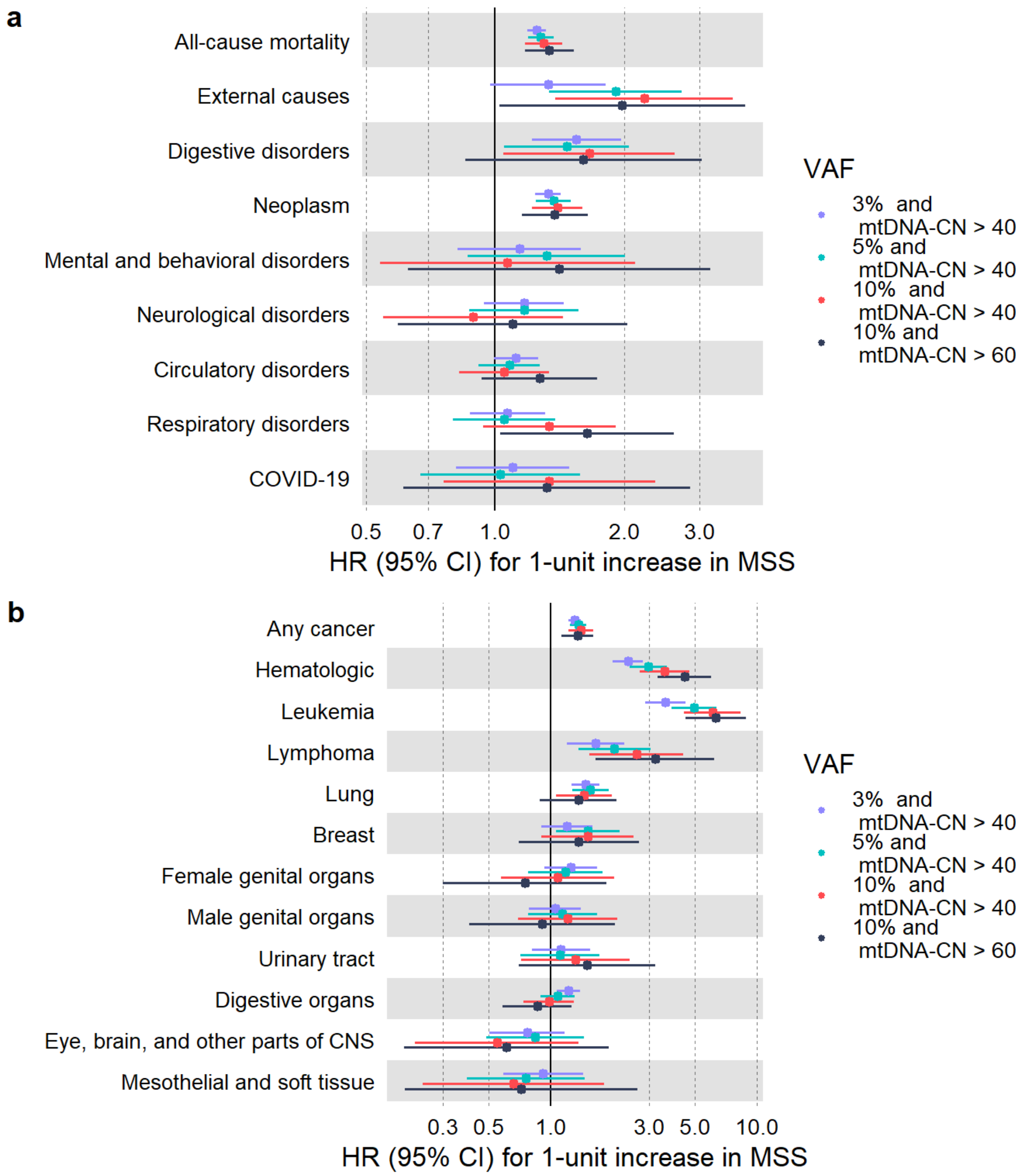

Supplementary Figure 9. Comparison of hazard ratios at different mitochondrial heteroplasmy thresholds. Hazard ratios (95% confidence intervals) for (**a**) each specific cause of death, and (**b**) cancer-specific mortality, were estimated using Cox proportional hazards models stratified by center and adjusted for age, sex, smoking status, alcohol intake, body mass index, white blood cell count, and haplogroup. Cases of death in fewer than 100 participants in any of the thresholds (deaths due to infection, benign diseases of the blood, endocrine disorders, or genitourinary disorders in (**a**) and deaths due to cancers of lip, oral cavity, and pharynx, thyroid and other endocrine glands, bone, or skin in (**b**)) are not included in the plot. Neoplasm in (**a**) included both benign and malignant neoplasms. Hematologic cancers included cancers of the lymphoid, hematopoietic, and related tissues. Lung cancers included cancers of the respiratory and intrathoracic organs. Abbreviations: CNS, central nervous system. The

number of events reflect the number of events in participants included in the regression analysis.

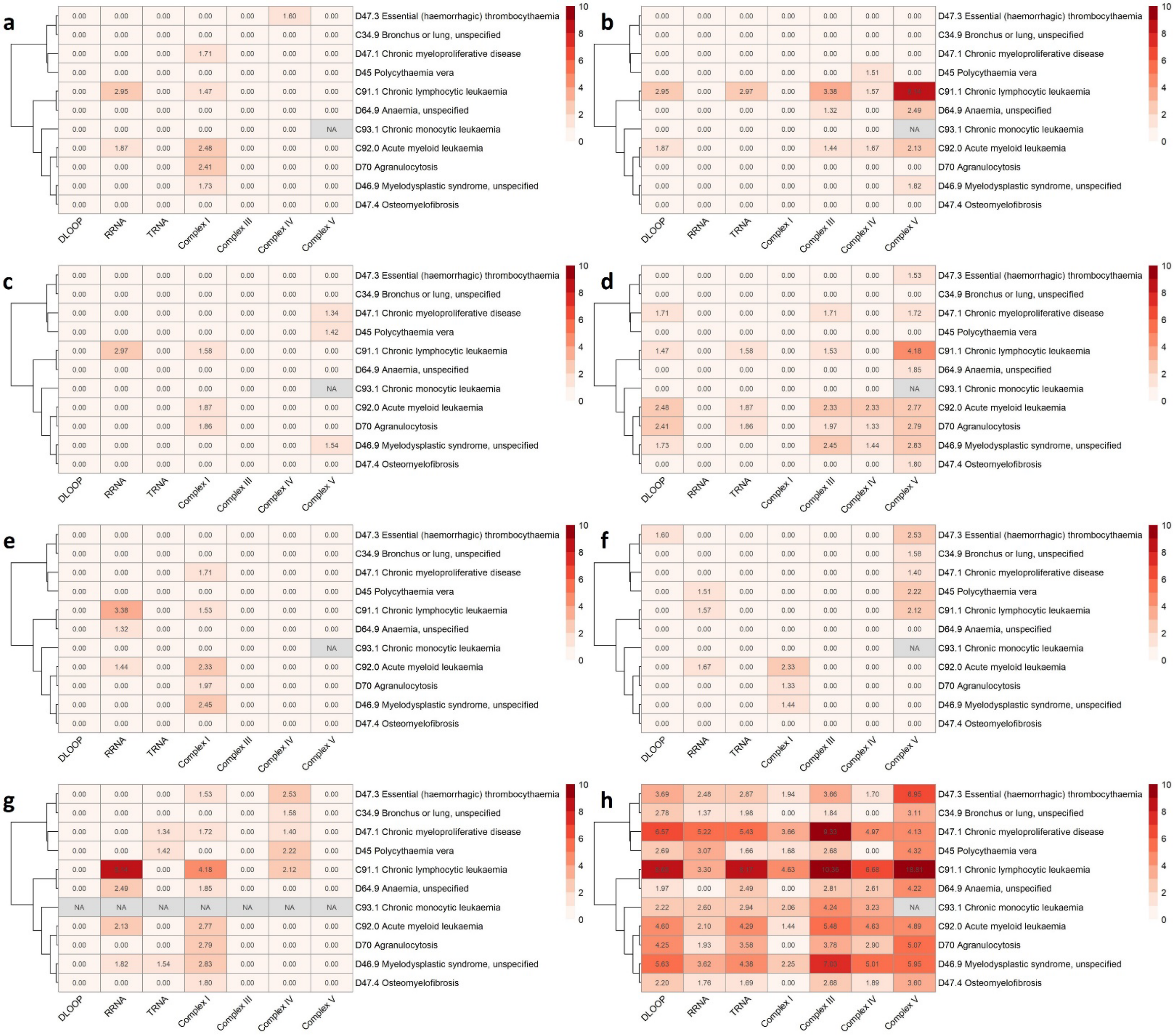
a
b
c
d
e
f
g
h
D47.3 Essential (haemorrhagic) thrombocythaemia
C34.9 Bronchus or lung, unspecified
D47.1 Chronic myeloproliferative disease
D45 Polycythaemia vera
C91.1 Chronic lymphocytic leukaemia
D64.9 Anaemia, unspecified
C93.1 Chronic monocytic leukaemia
C92.0 Acute myeloid leukaemia
D70 Agranulocytosis
D46.9 Myelodysplastic syndrome, unspecified
D47.4 Osteomyelofibrosis
DLOOP
RRNA
TRNA
Complex I
Complex III
Complex IV
Complex V

Supplementary Figure 10. Heatmap displaying the significance (-log10(P value)) of the difference between effect estimates for MSS and ICD 10 codes for cancer ("Chapter II Neoplasms") and hematological diseases ("Chapter III Disease of the blood and blood-forming organs and certain disorders involving the immune mechanism") by mtDNA region/complex. Effect estimates compared to estimates from (a) Dloop, (b) RRNA, (c) TRNA, (d) Complex I, (e) Complex III, (f) Complex IV, (g) Complex V, (h) All regions/complexes combined. ICD 10 codes were selected if the significance with the overall MSS was $<1x10^{-6}$, and clustered to match Fig. 8b. -log10(P values) >1.3 (corresponding to P values >0.05) were set to 0 for visualization.
