# Supplementary material for: Deleterious heteroplasmic mitochondrial mutations are associated with an increased risk of overall and cancer-specific mortality
Source: Nat Commun. 2023 Sep 30;14:6113. doi: 10.1038/s41467-023-41785-7 (PMC10542802; doi:10.1038/s41467-023-41785-7)
Supplement: Supplementary file 3 — Reporting Summary [file 41467_2023_41785_MOESM3_ESM.pdf]

nature portfolio

Corresponding author(s): Dan E. Arking

Last updated by author(s): Aug 7, 2023

Reporting Summary

Nature Portfolio wishes to improve the reproducibility of the work that we publish. This form provides structure for consistency and transparency in reporting. For further information on Nature Portfolio policies, see our Editorial Policies and the Editorial Policy Checklist.

Statistics

For all statistical analyses, confirm that the following items are present in the figure legend, table legend, main text, or Methods section.

n/a	Confirmed	
☐	☒	The exact sample size (*n*) for each experimental group/condition, given as a discrete number and unit of measurement
☐	☒	A statement on whether measurements were taken from distinct samples or whether the same sample was measured repeatedly
☐	☒	The statistical test(s) used AND whether they are one- or two-sided<br>*Only common tests should be described solely by name; describe more complex techniques in the Methods section.*
☐	☒	A description of all covariates tested
☐	☒	A description of any assumptions or corrections, such as tests of normality and adjustment for multiple comparisons
☐	☒	A full description of the statistical parameters including central tendency (e.g. means) or other basic estimates (e.g. regression coefficient) AND variation (e.g. standard deviation) or associated estimates of uncertainty (e.g. confidence intervals)
☐	☒	For null hypothesis testing, the test statistic (e.g. *F*, *t*, *r*) with confidence intervals, effect sizes, degrees of freedom and *P* value noted<br>*Give P values as exact values whenever suitable.*
☒	☐	For Bayesian analysis, information on the choice of priors and Markov chain Monte Carlo settings
☒	☐	For hierarchical and complex designs, identification of the appropriate level for tests and full reporting of outcomes
☐	☒	Estimates of effect sizes (e.g. Cohen's *d*, Pearson's *r*), indicating how they were calculated

Our web collection on statistics for biologists contains articles on many of the points above.

Software and code

Policy information about availability of computer code

Data collection	No software was used for data collection.
Data analysis	All code used to generate results, including data cleaning and data analysis, for this study can be found on the author's Github page: https://github.com/ArkingLab. Documentation on MitoHPC pipeline for DNA Nexus server is available in https://github.com/ArkingLab/MitoHPC/blob/main/docs/DNAnexus_CLOUD.md. Documentation on extracting Mitochondrial and NUMT reads from Google Cloud is available in https://github.com/ArkingLab/MitoHPC/blob/main/docs/GOOGLE_CLOUD.md. All analyses that were run in R were run in R v.4.3 (https://www.r-project.org) or Stata version 16.

For manuscripts utilizing custom algorithms or software that are central to the research but not yet described in published literature, software must be made available to editors and reviewers. We strongly encourage code deposition in a community repository (e.g. GitHub). See the Nature Portfolio guidelines for submitting code & software for further information.

Data

Policy information about availability of data

All manuscripts must include a data availability statement. This statement should provide the following information, where applicable:

- Accession codes, unique identifiers, or web links for publicly available datasets
- A description of any restrictions on data availability
- For clinical datasets or third party data, please ensure that the statement adheres to our policy

All data in this project were part of the UK Biobank resource and was accessed under application number 17731. Information on how to access the UK Biobank can be found at https://www.ukbiobank.ac.uk/enable-your-research/apply-for-access. TOPMed data are available through controlled access vis the NIH database of genotypes and phenotypes (dbGaP). Data for each participating study can be accessed through dbGaP with the corresponding accession numbers (ARIC, phs001211; FHS, phs000974; MESA, phs001416; and WHI, phs001237).

Research involving human participants, their data, or biological material

Policy information about studies with human participants or human data. See also policy information about sex, gender (identity/presentation), and sexual orientation and race, ethnicity and racism.

Reporting on sex and gender	In the UK Biobank, information on sex (Data Field ID 31) was based on central registry at recruitment but in some cases updated by the participant. The UK Biobank acknowledges that this variable may contain a mixture of the sex in the National Health Services had recorded for the participant and self-reported sex. We used the variable (Data Field ID 31) provided by the UK Biobank in our analysis. Of the 194,871 participants included in the final analysis, 87,225 (44.8%) were men and 107,646 (55.2%) were women. We have incorporated it as a covariate and also tested for interaction by sex in all our analysis. There were no major differences by sex and we report as such in the manuscript.<br>For TOPMed cohorts, we used self-reported sex and incorporated it as a covariate in the analysis.
Reporting on race, ethnicity, or other socially relevant groupings	For the analysis involving UK Biobank participants, we used self-reported race/ethnic background and grouped them into White, Black, Asian, and Other. We performed analyses stratified by race/ethnic background and report our findings in the manuscript. We also performed race-stratified analyses in TOPMed cohorts and report the results in combination with UK Biobank results using fixed-effects meta-analysis.
Population characteristics	he UK Biobank is a large population-based prospective study of 500,000 participants aged between 40 to 69 years recruited across the United Kingdom from 2006 to 2010. The UK Biobank collects extensive phenotypic and genotypic data on participants, which are used in our analysis. We included in our analysis 199,909 participants who underwent whole genome sequencing of the DNA from the blood draw and consented to be in the study. The mean age at the time of recruitment among those included in the study was 56.4 years (standard deviation 8.1) and 183,230 (94.0%) self-identified themselves as white. The covariates included in the main analyses were age, sex, smoking status (self-reported), alcohol intake (self-reported), body mass index, white blood cell count, and haplogroup. Haplogroup was grouped by phylogenetic similarly into the following: L is L0-L6; M is C, D, E, G, M, Q, Z; N is A, I, N, S, W, X Y; R is B, F, P, R; R0 is R0, HV, V; U is U, K; JT is JT, T; H is H only. The UK biobank is linked to national death registries and provides information on date of death and cause of death. Primary cause of death was coded using the ICD-10 codes and classified into 12 categories (infection [A00–B00, L00–L08]; neoplasm [C00–D48]; benign disease of the blood [D50–D89]; endocrine disorders [E00–E90]; mental and behavioral disorders [F00–F89]; neurological disorders [G00–G99]; circulatory disorders [I05–I89]; respiratory disorders [J09–J99]; digestive disorders [K20–K93]; genitourinary disorders [N00–N98]; COVID-10 [U07]; and external causes [V01–Y89]).<br>The UK Biobank also provides information on cancer diagnosis by linkage to national cancer registries, including type of cancer, date of cancer diagnosis, and age at cancer diagnosis. The type of cancer is coded using ICD-9 or ICD-10 codes, which we used to categorize into 15 types of cancer by organ system.
Recruitment	The UK Biobank is a large population-based prospective study of 500,000 participants aged between 40 to 69 years recruited across the United Kingdom from 2006 to 2010. The participants were invited for a visit to an assessment center for data collection, which included an automated questionnaire (on lifestyle factors, medical history, environmental factors, and cognitive function), physical examination, anthropometric measurements, and sampling of biospecimen (blood and urine samples) for laboratory tests. We included in our analysis 199,909 participants who underwent whole genome sequencing of the DNA from the blood draw and consented to be in the study. The UK Biobank participants are healthier than the general population of the UK, however, the association between mitochondrial DNA heteroplasmy and health outcomes is unlikely to be largely affected by the self-selection into the study, as previous studies have also shown expected associations of well-known risk factors with mortality. Moreover, the findings of our study have been validated in independent cohorts in the US
Ethics oversight	The UK Biobank was approved by the UK Biobank Research Ethics Committee and all participants provided written informed consent before participation. The current study was approved by the Johns Hopkins Medicine Institutional Review Boards.

Note that full information on the approval of the study protocol must also be provided in the manuscript.

Field-specific reporting

Please select the one below that is the best fit for your research. If you are not sure, read the appropriate sections before making your selection.

☒ Life sciences ☐ Behavioural & social sciences ☐ Ecological, evolutionary & environmental sciences

For a reference copy of the document with all sections, see nature.com/documents/nr-reporting-summary-flat.pdf

Life sciences study design

All studies must disclose on these points even when the disclosure is negative.

Sample size	A total of 194,871 participants with whole genome sequencing data and passed quality control procedures were included in the main analysis. The sample size was based on data availability and in all analysis, we used the largest sample size available at the time of analysis.
Data exclusions	We ran 200,000 WGS samples in the UK Biobank database through a newly developed pipeline, MitoHPC, to qualify mitochondrial DNA heteroplamsy. Of those 199,919 samples had outputs from MitoHPC variant calling and of those we calculated mtDNA copy number (mtDNA-CN) in 199,910 samples. MitoHPC outputs various metrics for assessing sample quality, allowing us to remove low quality samples prior to analysis. We excluded variants with read depth < 300 and those flagged as base quality, strandedness, slippage, weak evidence, germline, position flags in the FILTER column of the VCF. We further excluded heteroplasmic variants at poly-C homopolymer regions on the mitochondrial chromosome and excluded INDELs. We excluded samples based on a few, pre-defined criteria: potential mitochondrial contamination, 2 or more variants belonging to a different mitochondrial haplogroup, multiple variants predicted to be nuclear-encoded mitochondrial sequences (NUMTs), low minimum base coverage, and low mean base coverage which resulted in 2501 participants being excluded. Since mitochondrial heteroplasmy has previously been shown to be affected by low mtDNA-CN, we removed participants with mtDNA-CN less than 40 (n = 3580). Some samples met multiple exclusion criteria. We found that 358 participants had a heteroplasmic count above 5, with 175 of them identified as contaminated. We removed the remaining high heteroplasmic samples as these appeared to be outliers in our dataset with potentially unidentified contamination. The final sample size for downstream analysis was 194,871 participants.
Replication	Technical replications were performed by multiple sensitivity analyses (fitting alternative models), as described in the manuscript. In addition, we used 4 independent cohorts in the TOPMed program to validate the findings from the UK Biobank, which supported our findings.
Randomization	As an observational study, participants were not randomized into experimental groups.
Blinding	The authors had not been part of the recruitment, data acquisition, or data processing and, therefore, were blinded to the distribution of the exposure(s) and group allocation before performing data analysis.

Reporting for specific materials, systems and methods

We require information from authors about some types of materials, experimental systems and methods used in many studies. Here, indicate whether each material, system or method listed is relevant to your study. If you are not sure if a list item applies to your research, read the appropriate section before selecting a response.

Materials & experimental systems

n/a	Involved in the study	
☒	☐	Antibodies
☒	☐	Eukaryotic cell lines
☒	☐	Palaeontology and archaeology
☒	☐	Animals and other organisms
☒	☐	Clinical data
☒	☐	Dual use research of concern
☒	☐	Plants

Methods

n/a	Involved in the study	
☒	☐	ChIP-seq
☒	☐	Flow cytometry
☒	☐	MRI-based neuroimaging
