# Supplementary material for: Deleterious heteroplasmic mitochondrial mutations are associated with an increased risk of overall and cancer-specific mortality
Source: Nat Commun. 2023 Sep 30;14:6113. doi: 10.1038/s41467-023-41785-7 (PMC10542802; doi:10.1038/s41467-023-41785-7)
Supplement: Supplementary file 4 — Description of supplementary dataset [file 41467_2023_41785_MOESM4_ESM.docx]

**Description of Additional Supplementary Files**

Supplementary Data 1

Description: 60 unique pathogenic variants in the UK Biobank.

Supplementary Data 2

Description: A phenome-wide association study (PheWAS) results for association with MSS.

Supplementary Data 3

Description: PHESANT results for association with MSS.
